# A Smartphone-Based Model of Care to Support Patients With Cardiac Disease Transitioning From Hospital to the Community (TeleClinical Care): Pilot Randomized Controlled Trial

**DOI:** 10.2196/32554

**Published:** 2022-02-28

**Authors:** Praveen Indraratna, Uzzal Biswas, James McVeigh, Andrew Mamo, Joseph Magdy, Dominic Vickers, Elaine Watkins, Andreas Ziegl, Hueiming Liu, Nicholas Cholerton, Joan Li, Katie Holgate, Jennifer Fildes, Robyn Gallagher, Cate Ferry, Stephen Jan, Nancy Briggs, Guenter Schreier, Stephen J Redmond, Eugene Loh, Jennifer Yu, Nigel H Lovell, Sze-Yuan Ooi

**Affiliations:** 1 Department of Cardiology Prince of Wales Hospital Randwick Australia; 2 Prince of Wales Clinical School UNSW Sydney Sydney Australia; 3 Graduate School of Biomedical Engineering UNSW Sydney Sydney Australia; 4 Department of Cardiology The Sutherland Hospital Sydney Australia; 5 Center for Health and Bioresources Austrian Institute of Technology Graz Austria; 6 The George Institute for Global Health Sydney Australia; 7 Susan Wakil School of Nursing and Midwifery Charles Perkins Centre University of Sydney Sydney Australia; 8 National Heart Foundation of Australia Sydney Australia; 9 Stats Central Mark Wainwright Analytical Centre UNSW Sydney Sydney Australia; 10 School of Electrical and Electronic Engineering University College Dublin Dublin Ireland

**Keywords:** digital health, telemedicine, mHealth, heart failure, ischemic heart disease, mobile phone

## Abstract

**Background:**

Patients hospitalized with acute coronary syndrome (ACS) or heart failure (HF) are frequently readmitted. This is the first randomized controlled trial of a mobile health intervention that combines telemonitoring and education for inpatients with ACS or HF to prevent readmission.

**Objective:**

This study aims to investigate the feasibility, efficacy, and cost-effectiveness of a smartphone app–based model of care (TeleClinical Care [TCC]) in patients discharged after ACS or HF admission.

**Methods:**

In this pilot, 2-center randomized controlled trial, TCC was applied at discharge along with usual care to intervention arm participants. Control arm participants received usual care alone. Inclusion criteria were current admission with ACS or HF, ownership of a compatible smartphone, age ≥18 years, and provision of informed consent. The primary end point was the incidence of unplanned 30-day readmissions. Secondary end points included all-cause readmissions, cardiac readmissions, cardiac rehabilitation completion, medication adherence, cost-effectiveness, and user satisfaction. Intervention arm participants received the app and Bluetooth-enabled devices for measuring weight, blood pressure, and physical activity daily plus usual care. The devices automatically transmitted recordings to the patients’ smartphones and a central server. Thresholds for blood pressure, heart rate, and weight were determined by the treating cardiologists. Readings outside these thresholds were flagged to a monitoring team, who discussed salient abnormalities with the patients’ usual care providers (cardiologists, general practitioners, or HF outreach nurses), who were responsible for further management. The app also provided educational push notifications. Participants were followed up after 6 months.

**Results:**

Overall, 164 inpatients were randomized (TCC: 81/164, 49.4%; control: 83/164, 50.6%; mean age 61.5, SD 12.3 years; 130/164, 79.3% men; 128/164, 78% admitted with ACS). There were 11 unplanned 30-day readmissions in both groups (*P*=.97). Over a mean follow-up of 193 days, the intervention was associated with a significant reduction in unplanned hospital readmissions (21 in TCC vs 41 in the control arm; *P*=.02), including cardiac readmissions (11 in TCC vs 25 in the control arm; *P*=.03), and higher rates of cardiac rehabilitation completion (20/51, 39% vs 9/49, 18%; *P*=.03) and medication adherence (57/76, 75% vs 37/74, 50%; *P*=.002). The average usability rating for the app was 4.5/5. The intervention cost Aus $6028 (US $4342.26) per cardiac readmission saved. When modeled in a mainstream clinical setting, enrollment of 237 patients was projected to have the same expenditure compared with usual care, and enrollment of 500 patients was projected to save approximately Aus $100,000 (approximately US $70,000) annually.

**Conclusions:**

TCC was feasible and safe for inpatients with either ACS or HF. The incidence of 30-day readmissions was similar; however, long-term benefits were demonstrated, including fewer readmissions over 6 months, improved medication adherence, and improved cardiac rehabilitation completion.

**Trial Registration:**

Australian New Zealand Clinical Trials Registry ACTRN12618001547235; https://www.anzctr.org.au/Trial/Registration/TrialReview.aspx?id=375945

## Introduction

### Cardiovascular Disease

Cardiovascular disease remains the most prevalent cause of morbidity and mortality in high-income countries despite significant advances in treatment over the last 5 decades. Myocardial infarction is responsible for 15% of worldwide mortality [1], and heart failure (HF) affects >26 million people worldwide [2]. Recent epidemiological data show that cardiovascular mortality is no longer declining and is indeed rising in some communities [3], and hospitalization rates are universally increasing [4,5]. The principal drivers include an aging population and rising prevalence of adult and childhood obesity [6,7]. Coupled with increasing health care costs, these trends raise concerns regarding the sustainability of the already overburdened traditional model of health care.

Cardiac readmissions are a potential target for system improvement. Readmission rates for both acute coronary syndrome (ACS) and HF approach 20% for patients in the first month after discharge [8-10]. Readmissions are associated with increased mortality and costs for the health care system [11]. In Australia, the estimated annual cost of readmissions for HF exceeds Aus $600 million (US $463.7 million) [12]. A recent audit of 3 hospitals in the state of New South Wales reported that 27% of angina pectoris admissions and 63% of HF admissions were preventable [13].

Up to 45% of mortality from recurrent myocardial infarction is preventable [14]. Secondary prevention for both conditions (ACS and HF) is critical and involves maximizing medication compliance, self-care, and optimization of modifiable risk factors, including weight and blood pressure (BP). However, secondary prevention programs have suboptimal uptake. For ACS, the cornerstone of secondary prevention is cardiac rehabilitation (CR), which is only attended by 20% to 30% of eligible participants because of competing demands such as employment and family responsibilities as well as travel time and costs [15]. For HF, management using community HF teams is resource-intensive and not uniformly available.

### Telehealth

Telehealth, the provision of health care by means of telecommunication technology, is a valuable adjunct in the management of chronic diseases. Within the scope of telehealth is mobile health (mHealth), which uses ubiquitous mobile phone technology for service delivery. Broadly, mHealth interventions encompass SMS text messaging strategies and telemonitoring systems in the form of smartphone apps. Telemonitoring is the practice of remote transmission and receipt of physical parameters such as pulse rate, BP, and weight. A recent meta-analysis found that the use of mHealth interventions in cardiovascular disease was associated with an improvement in BP and HF hospitalization rates [16]. The most successful interventions included several key factors: a method of *flagging* abnormal results, involvement of the patients’ usual health care providers, and automatic data transmission as opposed to manual data entry by the patients. Thus, from a collaboration between a team of hospital-based clinicians and biomedical engineers, the TeleClinical Care (TCC) smartphone app was developed to include all these factors. Crucially, the app contains an educational component in addition to telemonitoring, making it a rare multifunctional mHealth intervention to undergo a randomized controlled trial (RCT). The app was designed to be used by patients diagnosed with either ACS or HF to maximize uptake. It is the first mHealth telemonitoring intervention to be trialed in Australian patients with HF.

### Objectives

The primary objective is to examine the efficacy of the TCC model compared with usual care alone on the incidence of 30-day hospital readmission rates in patients recently discharged with ACS or HF. Secondary objectives include: (1) to describe the compliance rate with the intervention as well as the frequency of alerts and actions subsequently undertaken, (2) to examine the impact of the intervention on clinical outcomes, (3) to examine the cost-effectiveness of the intervention, and (4) to measure patient satisfaction with the intervention.

## Methods

### Participants

Patients were recruited between February 2019 and March 2020 from 2 hospitals in Sydney, New South Wales, Australia (Prince of Wales Hospital and The Sutherland Hospital). Patients were eligible if they were being discharged after an admission for either HF or ACS, were aged ≥18 years, and owned a compatible smartphone (defined as operating either Apple iOS 9.0 or above, or Android 7.0 Nougat or above). The exclusion criteria were inability or unwillingness to provide informed consent, inability to operate the app because of physical or cognitive limitations, inability to attend in-person follow-up (such as participants who normally resided outside of Sydney) or travel overseas for any duration within the first 30 days after discharge or for a period of >1 month, or expected discharge to another hospital or a nursing home. Advanced age, comorbidities, and familiarity with smartphone apps were not used as inclusion or exclusion criteria. All patients who met the inclusion criteria were approached for participation. The participants did not receive any financial compensation during the trial.

### Ethics

This study received ethical approval from the South Eastern Sydney Local Health District Human Research Ethics Committee (approval number 2019/ETH11442). The study was registered with the Australian New Zealand Clinical Trials Registry (ACTRN12618001547235).

### Enrollment

Patients were enrolled during the index admission after providing written informed consent. All baseline data were collected before discharge and before randomization. BP and weight were measured using an automatic digital sphygmomanometer (A&D Medical UA-651BLE) and a digital weighing scale (A&D Medical UC-352BLE). These same devices were provided to the participants assigned to the intervention arm. BP was measured in the seated position. A total of 2 measurements were taken 1 to 5 minutes apart and averaged. If the 2 systolic readings differed by >15 mm Hg, a third measurement was taken, and the 2 closest readings were averaged. Height was measured using a wall-mounted stadiometer. Waist circumference was measured halfway between the costal margin and iliac crest as per World Health Organization guidelines [17]. A 6-minute walk distance test was performed using a graduated 25-meter track with standardized encouragement according to the protocol described by the Lung Foundation of Australia [18]. The test was not performed on those unsafe to complete it because of frailty, unsteadiness, or physical limitations. Serum low-density lipoprotein cholesterol levels were measured in blood samples previously obtained during hospitalization. A written questionnaire was provided to the participants containing the Morisky–Green–Levine 4-item medication compliance (MGL) score [19], the 5-level EuroQol 5-dimension quality of life assessment [20], and the Patient Activation Measure [21]. All baseline data were collected by study investigators: PI and JMc at Prince of Wales Hospital, and AM and JM at Sutherland Hospital.

### Randomization

Before discharge, the participants were randomized 1:1 into either TCC plus usual care or usual care alone. Block randomization was performed using a randomization schedule created by an independent statistician, which was subsequently deployed within a web-based system (Research Electronic Data Capture) [22]. Randomization strata included hospital and primary diagnosis (ACS or HF). Randomization was performed by the investigator who collected the baseline data (PI and JMc at Prince of Wales Hospital, and AM and JM at Sutherland Hospital).

### Intervention

**Figure 1:**
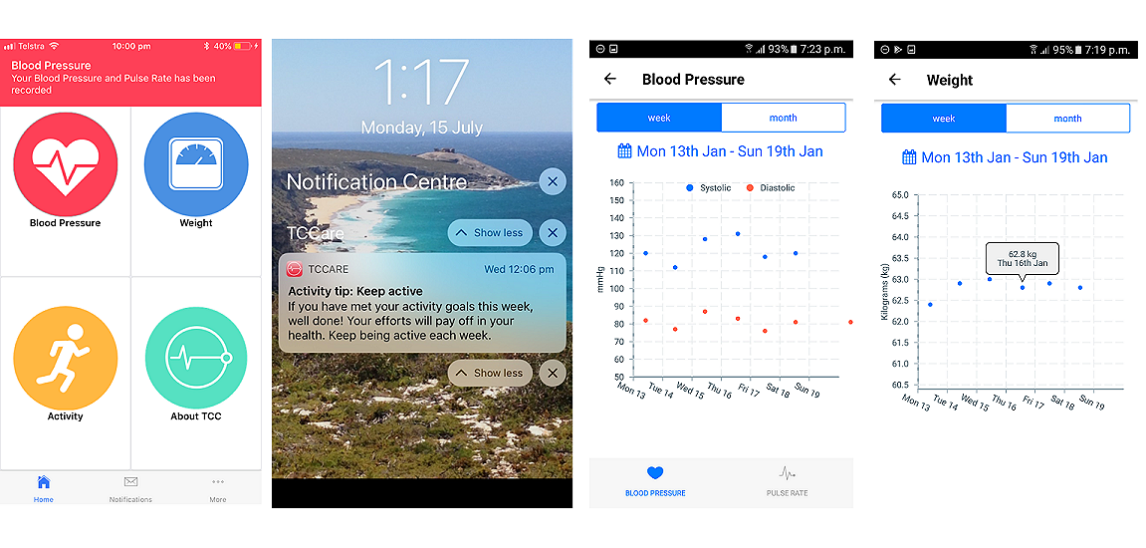
Screenshots of the TeleClinical Care (TCC) app. From left to right: the TCC app home screen, the appearance of an educational notification, weekly record of blood pressure readings, and weekly record of weight readings.

**Figure 2:**
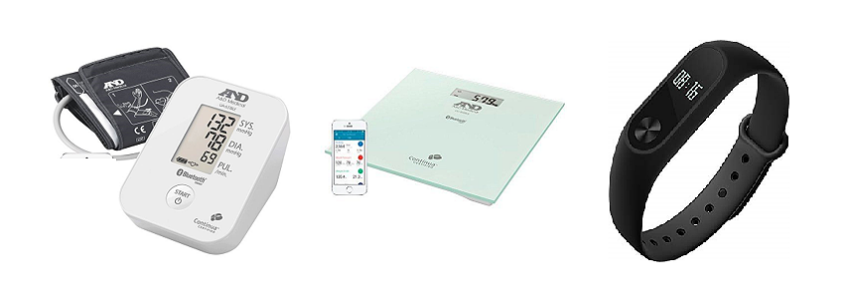
Bluetooth-enabled peripheral devices. From left to right: sphygmomanometer (A&D Medical UA-651BLE), weighing scale (A&D Medical UC-352BLE), and activity monitor (Xiaomi MiBand 2).

**Figure 3:**
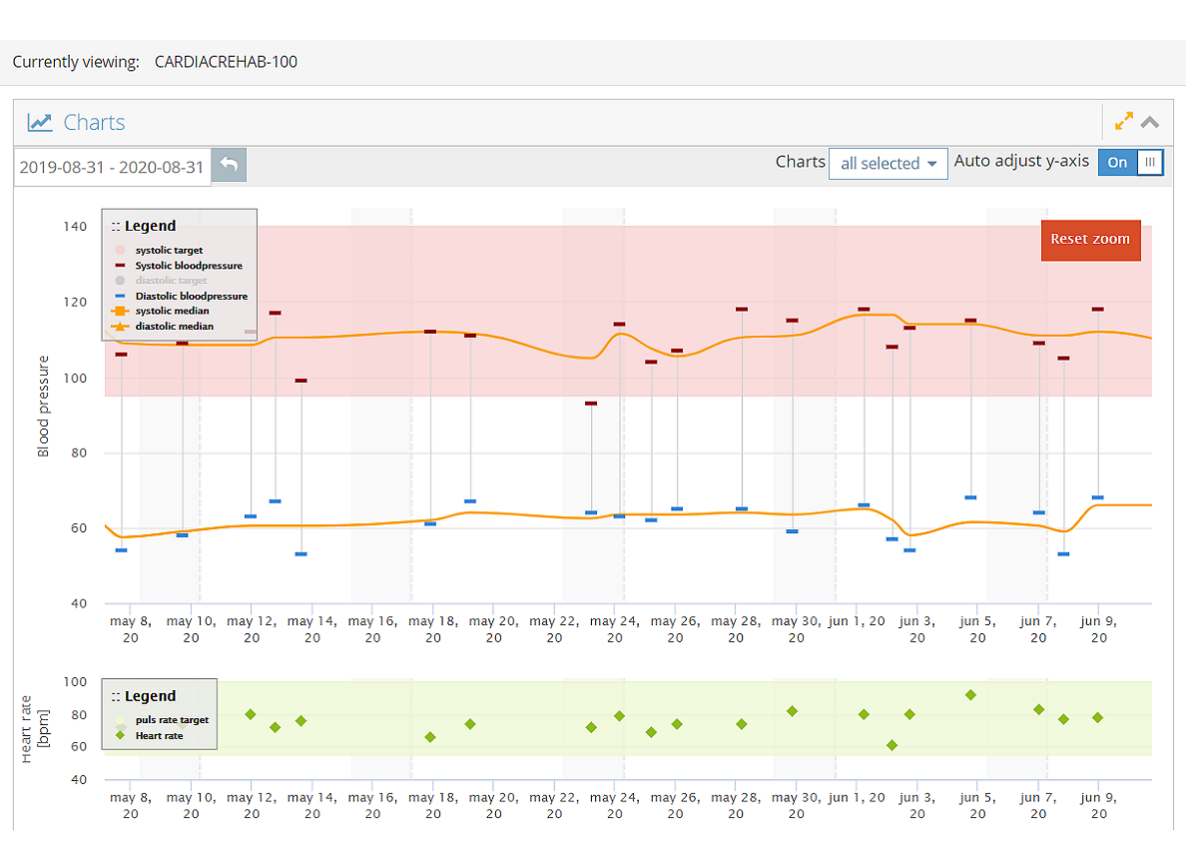
Screenshot of the KIOLA back-end, which is visible to monitoring clinicians. Blood pressure and pulse rate are recorded when the data are sent from the Bluetooth-enabled sphygmomanometer. Readings outside the shaded zone automatically trigger an email alert to the monitoring clinicians. Bpm: beats per minute.

The participants assigned to the intervention arm received the TCC app (Figure 1) on their smartphone and connected Bluetooth peripheral devices at the time of discharge: a digital sphygmomanometer, a digital weighing scale, and a fitness band (Xiaomi MiBand 2; Figure 2). The participants were instructed to measure BP and pulse rate via the sphygmomanometer, as well as weight, daily. Before discharge, the participants were shown how to use the devices and performed 1 measurement with each device under the supervision of the research team member to ensure the correct technique. The participants were also provided with a pamphlet that described the correct technique for using the devices and some basic troubleshooting advice. Activity data were obtained either via the smartphone or the fitness band as minutes of activity per day. Readings could be performed at any time relative to medication dosing. Readings were automatically transmitted from the peripheral devices to the smartphone app via Bluetooth and subsequently to a web-based server (KIOLA; Figure 3) developed at the Austrian Institute of Technology and adapted for the Australian context by the technical members of our team. Readings could be displayed within the app in graphical form for viewing by the patient. These graphs could be presented to the patient’s general practitioner (GP) or cardiologist at follow-up visits, but this was not mandated. The app provided 3 weekly educational push notifications to promote healthy behavior choices, including dietary advice, physical exercise, and smoking cessation. The text for these notifications was based on the National Heart Foundation of Australia’s *Managing My Heart Health* consumer resource [23].

If readings had not been received by the server for >48 hours, the participant was contacted by a biomedical engineer to ascertain if there had been a technical issue. If the patient admitted noncompliance with the program on 3 separate occasions, they were not contacted again for absent readings.

For each patient, customizable limits for BP, pulse rate, and weight gain were defined at the time of discharge in consultation with the treating cardiologist. This was a 2-tier system of *yellow* (low priority) and *red* (high priority) alerts. For example, for a particular patient, a systolic BP >180 mm Hg could be defined as a red alert, and a systolic BP of 160-179 mm Hg could be defined as a yellow alert. The limits could be modified during the trial at the discretion of the monitoring team. If a reading returned outside of the defined limits, an alert was delivered by email to the monitoring team, which consisted of a cardiologist and a cardiac nurse practitioner who alternated monitoring duties. Emails were monitored from 8 AM to 5 PM on weekdays. Alerts delivered after hours, on weekends, or on public holidays were assessed the following weekday. Upon reviewing an alert, the monitoring clinician would decide whether to contact the patient and, upon doing so, assess whether the alert required escalation to the patient’s GP or cardiologist. Patients were mandatorily contacted following receipt of any red alert. For yellow alerts, the monitoring team contacted the patients based on their own discretion. For example, alerts that were clearly erroneous (eg, a weight reading of 150 kg in a patient who normally weighed 75 kg) or those that rapidly normalized or were only marginally above the threshold and not considered clinically significant did not mandatorily require patient contact. Decisions to alter management or order investigations were made by the patient’s GP or cardiologist and not by the monitoring team. All alerts, response details, and outcomes were recorded. Usual care, provided in both arms, included a recommendation to follow up with the GP within 1 week of discharge and with the treating cardiologist, who determined the timing of this visit. Patients with ACS were referred to CR, and patients with HF were referred to the local HF outreach service.

### Outcome Parameters

The participants were followed up at 6 months. This occurred in person until March 2020 and then by telephone after COVID-19 was defined as a global pandemic. The primary outcome was the number of readmissions at 30 days, which was chosen because early readmissions are designated as a key hospital performance indicator by the state government. The occurrence of readmissions as well as the length of stay were confirmed by patient interviews, review of the local electronic medical records, and the Australian national health database (MyHealthRecord). A readmission was defined as an unplanned return to hospital, either via the emergency department (ED) or direct admission, resulting in the acceptance of care of the patient by any inpatient medical team. Planned admissions, ED presentations that resulted in discharge without inpatient admission, and admissions to the ED short stay unit were not considered readmissions for the purpose of this study. The cause of the readmission was determined by the summary diagnosis given in the discharge summary and was classified as noncardiac or cardiac. CR attendance was defined as presence during at least one session. CR completion was defined as attendance to ≥10 sessions or formal discharge by the CR staff. CR attendance was routinely recorded in the patient’s electronic medical record by the CR staff at both hospitals. Only patients with ACS were included in this analysis as patients with HF are not routinely referred to CR at either institution. The analysis was limited to those enrolled ≥2 months before the closure of CR for COVID-19. For in-person visits, physical parameters were measured by blinded investigators. During the COVID-19 pandemic, the final BP (average of the last 2 readings) and weight were obtained from the readings submitted via the app for those in the intervention arm; however, the corresponding values were not obtained from the participants in the control arm. Follow-up blood tests were not mandated during the pandemic. The participants completed the same questionnaires at baseline by either written or telephone means depending on whether the follow-up date was before or during the pandemic. The participants in the intervention arm completed an evaluation of the TCC program (user experience questionnaire) in either written, telephone, or web-based form (Multimedia Appendix 1). This questionnaire was designed specifically for this study. Alerts were defined as clinically significant alerts if they led to a change in investigation or management or led directly to a consultation with a health care professional. Major adverse cardiovascular events were defined as a composite of all-cause death, nonfatal myocardial infarction, and nonfatal stroke.

### Statistical Analysis

As this was a pilot study, the sample size was not determined by a formal power calculation. Readmission analysis was performed using the Andersen–Gill Cox regression model. Single continuous variables were analyzed using the 2-tailed *t* test. Repeated measures were analyzed using linear mixed models. Nonparametric variables were analyzed using the Mann–Whitney *U* test. Single categorical variables were analyzed using the Pearson chi-square test. Repeated categorical variables were analyzed using generalized linear mixed models. Linear and generalized linear mixed models generated both a time interaction (change in parameters from baseline to follow-up) and a group-by-time interaction (change in parameters over time and between groups). Statistical analysis was performed using Stata Statistical Software: Release 16 (StataCorp LLC) and IBM SPSS Statistics for Windows, version 26.0. All analyses applied the intention-to-treat principle.

### Cost-Effectiveness

Running costs were recorded over the duration of the trial. Components of the running costs included the cost of equipment, staffing costs, server maintenance costs, and the cost of health care consultations generated by the system. A figure of cost per cardiac readmission saved was calculated by dividing the total cost incurred by the difference in cardiac readmission rates between the 2 groups. As costs in the research setting were unlikely to reflect mainstream clinical practice, a 12-month *real world* cost-effectiveness model was undertaken (Multimedia Appendix 2).

## Results

### Screening, Enrollment, and Follow-up

**Figure 4:**
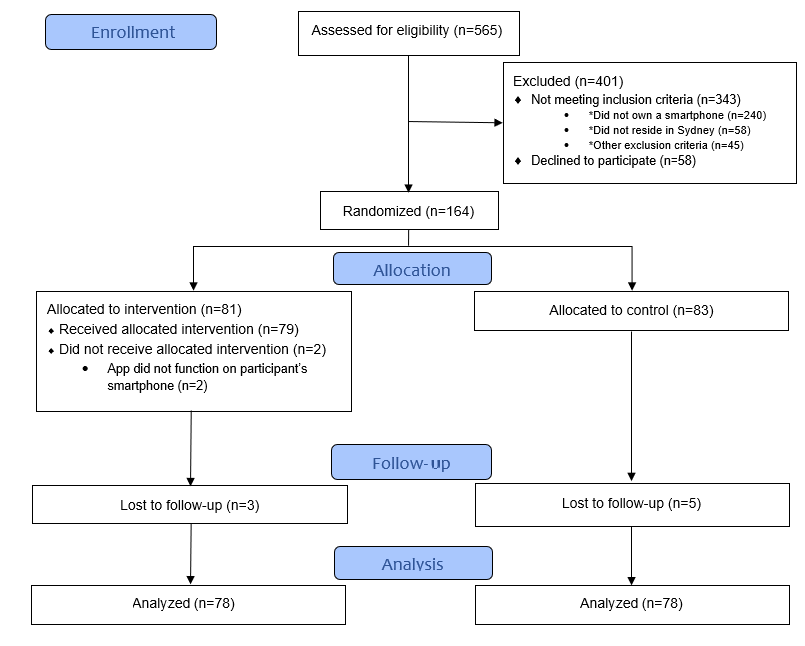
Enrollment flowchart.

Between February 2019 and March 2020, 565 potential participants were screened for eligibility, of which 240 (42.5%) did not own a compatible smartphone, which was the most common reason for exclusion. Approximately 28.5% (161/565) of patients met ≥1 of the remaining exclusion criteria, the most common reasons being unwillingness to participate and living outside Sydney (and being unable to return for in-person follow-up; 58/161, 36% of patients in each case). A total of 128 patients with ACS and 36 patients with HF were enrolled for a total of 164 participants (Figure 4). Enrollment was terminated early at the onset of the COVID-19 pandemic. The app did not operate on the smartphones of 2 patients in the intervention arm (2/164, 1.2%). Another patient chose not to use the app after randomization because of a new diagnosis of lung cancer, although he did not withdraw from the study. These 3 patients (3/164, 1.8%) and all others randomized into the intervention regardless of compliance were included as part of the intention-to-treat analysis. The mean follow-up time was 193 days. Of the 164 patients, 8 (4.9%) were lost to follow-up.

### Baseline Characteristics

The mean age was 61.5 years, and 79.3% (130/164) of patients were men (Table 1). Approximately 25.6% (42/164) of patients had moderate or severe left ventricular dysfunction. Most patients received guideline-directed medical therapy at baseline (Multimedia Appendix 3).

**Table 1:** Baseline characteristics of the enrolled cohort (N=164).

Characteristic			TCC^a^ (n=81)		Control (n=83)
Age (years), mean (SD)			61.3 (12.3)		61.7 (12.6)
**Gender, n (%)**					
	Male	65 (80)		65 (78)	
	Female	16 (20)		18 (22)	
**Clinical characteristics**					
	ACS,^b^ n (%)	63 (78)		65 (78)	
	HF,^c^ n (%)	18 (22)		18 (22)	
	Moderate or severe LV^d^ dysfunction, n (%)	21 (26)		21 (25)	
	Current smoker, n (%)	18 (22)		21 (25)	
	Atrial fibrillation, n (%)	16 (20)		20 (24)	
	Hypertension, n (%)	39 (48)		46 (55)	
	Diabetes, n (%)	21 (26)		22 (27)	
	Chronic kidney disease, n (%)	11 (14)		12 (14)	
	Systolic BP^e^ (mm Hg), mean (SD)	119 (18)		121 (18)	
	Weight (kg), mean (SD)	85.0 (16.8)		87.9 (22.3)	
	BMI (kg/m^2^), mean (SD)	28.5 (4.5)		30.1 (6)	
	Waist circumference (cm), mean (SD)	100 (13)		104 (16)	
	6-minute walk test distance (m), mean (SD)	385 (119)		353 (124)	
	LDL-C^f^ (mmol/L), mean (SD)	2.33 (0.9)		2.26 (1.05)	
5-level EuroQol 5-dimension calculated score (−0.10 to 1.00), mean (SD)			0.84 (0.17)		0.80 (0.17)
Self-reported quality of life score (0-100), mean (SD)			66.7 (18)		63.1 (21)
MGL^g^ score (0-4)			3.11		3.29
Patient Activation Measure (0-100), mean (SD)			64.5 (15)		63 (16)

^a^TCC: TeleClinical Care.

^b^ACS: acute coronary syndrome.

^c^HF: heart failure.

^d^LV: left ventricular.

^e^BP: blood pressure.

^f^LDL-C: low-density lipoprotein cholesterol.

^g^MGL: Morisky–Green–Levine 4-item medication compliance.

### Readmissions at 30 Days

All-cause, unplanned readmissions at 30 days were similar in the 2 groups (11 in the intervention arm and 11 in the control arm; *P*=.97).

### Total Readmissions

**Figure 5:**
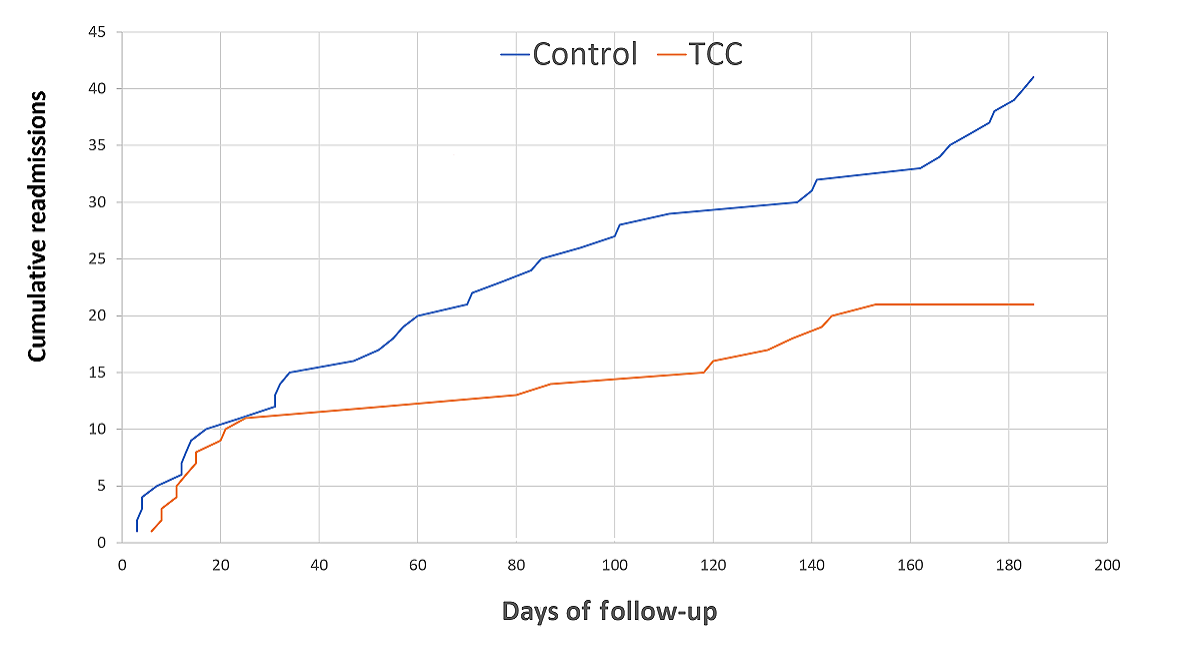
Cumulative readmissions over the course of the trial. TCC: TeleClinical Care.

At 6 months, the intervention was associated with a reduction in all-cause, unplanned readmissions, with a total of 21 readmissions in the intervention arm and 41 readmissions in the control arm (hazard ratio [HR] 0.51, 95% CI 0.31-0.88; *P*=.02; Figure 5). Cardiac readmissions were also less common in the intervention arm (11 in the intervention arm vs 25 in the control arm; HR 0.44, 95% CI 0.22-0.90; *P*=.03). There was a numeric reduction in noncardiac readmissions during the study period, which did not reach statistical significance (10 in the intervention arm vs 16 in the control arm; HR 0.64, 95% CI 0.29-1.40; *P*=.26)*.* Among patients with HF, there were 5 cardiac readmissions in the intervention arm and 18 readmissions in the control arm; however, this difference did not reach statistical significance, likely because of the smaller patient population.

### Compliance With the Intervention

The 2 patients for whom the app did not function on their smartphones were excluded from the compliance analysis. The average percentage of days that the participants transmitted data was 64.2% (SD 27.5%). BP and weight transmissions occurred at equal frequencies (64.2% of days each). Of the 79 patients, 60 (76%) transmitted data on >50% of days. Approximately 52% (42/81) of patients transmitted data on an average of ≥5 days per week (ie, more than 71% of all days). Approximately 20% (16/79) of patients transmitted data for less than an average of 3 days per week.

### Alerts

A total of 585 (2.5%) alerts were generated out of 23,401 transmissions, of which 419 (71.6%) were for the 63 patients with ACS (mean 6.7 alerts per patient), and 166 (28.4%) were for the 18 patients with HF (mean 9.2 alerts per patient; Multimedia Appendix 4). Of the 79 patients, 11 (14%) did not generate any alerts. On the basis of their interpretation of the alerts, the monitoring clinicians chose to contact patients after 30.9% (181/585) of alerts, with a mean and median response time of 12.5 hours and 5.0 hours, respectively. Approximately 12.5% (73/585) of alerts required discussion with one of the patient’s health care professionals. The remaining alerts were either erroneous, rapidly normalized, or not of clinical concern (Multimedia Appendix 4). A total of 54 health care consultations were generated from the alerts. The timing of 83% (45/54) of these consultations was known, and the mean time to consultation was 56 hours (median 26 hours). Approximately 16.1% (94/585) of alerts were clinically significant*.* Of the 79 patients, 42 (53%) did not generate any clinically significant alerts. The causes of the alerts can be found in Multimedia Appendix 4.

### Clinical Outcomes

A total of 4 deaths occurred in the control arm (4/83, 5%) and 1 in the intervention arm (1/81, 1%). All deaths were of cardiovascular causes. There was 1 nonfatal myocardial infarction in the intervention arm (1/81, 1%) and none in the control arm. No strokes occurred in either group. There was no statistically significant difference in mortality or major adverse cardiovascular events (Table 2).

**Table 2:** Major adverse cardiovascular events (MACEs; N=164).

Clinical outcome	TCC^a^ (n=81)	Control (n=83)	Relative risk (95% CI)	*P* value
Mortality	1	4	0.25 (0.03-2.24)	.22
Nonfatal MI^b^	1	0	3.07 (0.13-74.3)	.49
Nonfatal stroke	0	0	—^c^	—
MACEs	2	4	0.51 (0.10-2.72)	.43

^a^TCC: TeleClinical Care.

^b^MI: myocardial infarction.

^c^Not possible to calculate as there were no events.

### CR Attendance and Completion

There was no significant difference in CR attendance rates. However, there were statistically significant differences in CR completion rates, both as a proportion of participants who attended and as a proportion of the total group population (Table 3).

**Table 3:** Cardiac rehabilitation completion rates (N=100).

Parameter	TCC^a^ (n=51), n (%)	Control (n=49), n (%)	Statistical analysis	
			OR^b^ (95% CI)	*P* value
Attendance rate	28 (55)	21 (43)	1.62 (0.74-3.58)	.23
Completion rate (attendees only)	20 (71)^c^	9 (43)^d^	3.30 (1.01-11)	.04
Completion rate	20 (39)	9 (18)	2.90 (1.15-7.17)	.02

^a^TCC: TeleClinical Care.

^b^OR: odds ratio.

^c^n=28.

^d^n=21.

### Physical Parameters

Because of the cancellation of in-person visits, these outcomes could not be assessed for many participants. The results are summarized in Multimedia Appendix 5.

### Questionnaire Results

At baseline, 10 patients in the intervention arm (10/81, 12%) and 14 in the control arm (14/83, 17%) did not use regular medications. These patients did not complete the MGL questionnaire at baseline but were instructed to complete it at follow-up. The proportion of patients who reported good adherence (defined by an MGL score of 4/4) improved significantly in the intervention arm (34/71, 48% to 57/76, 75%; *P*<.001)*.* In the control arm, this proportion fell from 61% (42/69) to 50% (37/74; *P*=.19). Overall, there was a significant interaction favoring the intervention arm (*P*=.002).

The self-reported quality of life score from the 5-level EuroQol 5-dimension questionnaire improved significantly in both groups, but there was no difference between groups. The Patient Activation Measure score improved significantly in both groups, but there was no difference between groups (Multimedia Appendix 6).

### User Experience

Of the 81 participants, 66 (81%) completed the questionnaire. Reasons for noncompletion included limited use of the app, inadequate understanding of English, or declining to participate. The average rating out of 5 given for the app was 4.56. Approximately 96% (64/67) of users rated it as *easy* or *very easy* to use.

### Cost-Effectiveness

#### Trial Costs

The trial ran for 20 months. Staffing costs were Aus $53,435 (US $38,491.80), which comprised total remuneration for staff responsible for enrolling and monitoring of participants. Technical support was provided as in-kind support. Equipment for the 81 participants in the intervention arm had a total cost of Aus $18,630 (US $13,420.10), and server maintenance costs totaled Aus $9000 (US $6483.14). The trial generated 18 additional GP visits with a total cost of Aus $698 (US $502.80) and 17 cardiologist visits with a total cost of Aus $1343 (US $967.43). HF outreach services do not have a defined per-visit cost; thus, no additional costs were included. Thus, we calculated the total cost of the intervention as Aus $82,408 (US $59,362.50). There were 14 fewer cardiac readmissions in the control arm, which was adjusted to 13.67 given the slightly higher number of patients in the control arm. Thus, the cost per cardiac readmission saved was Aus $6028 (US $4342.26).

In the control arm, the total cost of cardiac readmissions for all patients combined was Aus $85,213 (US $61,383). In the intervention arm, the equivalent cost was Aus $38,640 (US $27,834.30). The reduction in costs from readmission avoidance was Aus $550 (US $396.19) per patient for a 6-month participation, which was doubled to Aus $1100 (US $792.38) for the projected 12-month real-world model.

#### Model for 12 Months

**Figure 6:**
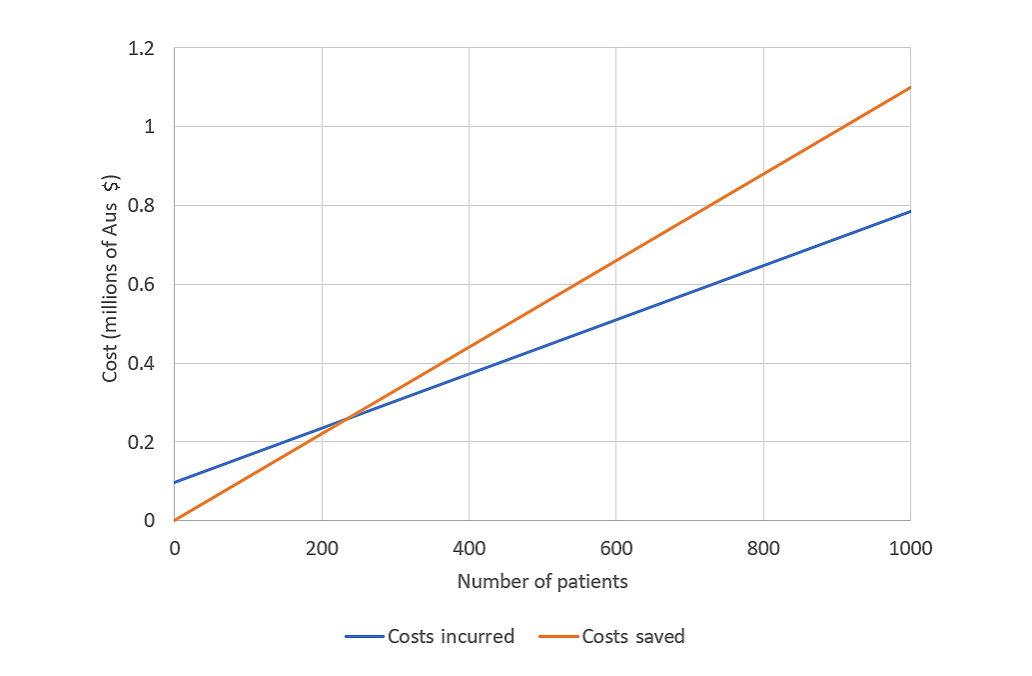
Cost-Effectiveness of the TeleClinical Care model as described by total costs incurred by the system and total costs saved by projected cardiac readmission prevention. The x-axis represents the number of patients enrolled, and the y-axis represents the cost in millions of Aus $.

In this model, it was calculated that each patient would require approximately 5.8 hours of attention from the monitoring team if enrolled for 12 months (Multimedia Appendix 7). The standard per-hour nursing cost was Aus $49.85 (US $35.91), thus generating a per-patient nursing cost of Aus $289 (US $208.18). For a single nurse working 40 hours per week, it was estimated that they could simultaneously monitor up to 358 patients. Equipment costs were revised to Aus $200 (US $144.07) per patient as the MiBand 2 activity monitor was not intended for future use. Costs generated because of medical consultations were doubled to reflect a 12-month participation, as were costs saved by avoiding readmissions. Outside of the research setting, technical costs were projected to be higher because of the requirement of a commercial license for the KIOLA platform and technical support. A baseline cost of Aus $92,388 (US $66,551.50) was applied, as well as an annual cost of Aus $184 (US $132.54) per patient. A graph of the cost-effectiveness model is shown in Figure 6. According to this model, when the number of enrolled patients is ≥237, the costs saved from the prevention of readmissions will surpass all incurred costs.

## Discussion

### Principal Findings

The TCC program, which combined telemonitoring and educational messaging within a smartphone app, was not associated with a reduction in readmissions at 30 days in patients discharged after an admission with ACS or HF, although event rates were low, particularly for cardiac readmissions. However, the intervention showed benefit with respect to reducing the incidence of cardiac and all-cause readmissions over the 6-month study period, as well as an improvement in CR completion rate and medication compliance.

There is a paucity of data for mHealth interventions targeting patients with ACS or a general inpatient population such as the one examined in this study. Telemedical interventions for HF have yielded varying results, which can be explained by the heterogeneous nature of the interventions. A variety of protocols and patient populations have been previously described and, therefore, drawing parallels between trials is often troublesome.

A large early trial, *Tele-HF* (2010), used a voice-interactive system and included body weight as the sole physical parameter without measurement of pulse or BP [24]. Compliance was poor, with approximately only 50% of participants taking part ≥3 times per week. This trial was negative for its primary end point (all-cause death or readmission within 180 days of enrollment) but demonstrated possible improvements for future telemedicine systems.

An example of how varying intervention design and delivery may influence results is seen by comparison of the Telemedical Interventional Monitoring in Heart Failure (*TIM-HF*) [25] and *TIM-HF2* [26] trials, both conducted by the same German group. In the *TIM-HF*, 710 stable outpatients were enrolled, and electrocardiogram, BP, and body weight results were transmitted via a PDA and mobile phone service where they were reviewed by an independent clinician who communicated with the patients’ usual practitioner every 3 months. No difference in mortality or readmission was observed. In the *TIM-HF2,* a study of 1571 patients, a similar system was applied but with the addition of regular interaction with the patient’s GP and cardiologist. Here, a reduction in percentage days lost because of cardiovascular readmissions and all-cause death was observed (4.88% vs 6.64%; *P*=.046). The authors identified the ability to guide the patient’s management through their usual provider as a contributor to the success of the trial.

In comparison with these interventions, TCC had several advantages. The participants were required to measure BP, weight, and activity daily and, unlike several other mHealth studies in patients with cardiac disease [27-29], the results were automatically transmitted, thus removing the need for participants to manually enter data, which is burdensome and potentially error-prone. By aiming for daily transmission, there was a significant volume of data to detect trends in readings and to contextualize abnormalities. Weekly data entry, as has previously been described [29], is unlikely to be sensitive enough to detect clinical deterioration and thus prevent readmissions.

The alerts were automated, which allowed the monitoring team to efficiently identify the patients that required attention. The monitoring team, which consisted of a cardiologist and cardiac nurse practitioner, had significant clinical experience and was comfortable in deciding which alerts were clinically significant and which were not. As a result, <10% of all alerts received (54/585, 9.2%) led directly to a health care consultation. The involvement of the patients’ usual health care providers was also important as it is assumed that knowledge of the patient and their medical background is key to the interpretation and management of alerts. To date, only 1 individual mHealth RCT has demonstrated a reduction in readmissions in patients with HF. This study by Dendale et al [30] randomized 160 patients with HF and similarly used automated data transmission combined with interaction with the patients’ usual health care providers. On average, the patients generated 27 alerts over the 6-month period, which was higher than what was observed in this study.

Compliance with TCC was reasonable, with participants transmitting data on approximately 64% of study days. This is lower than in other mHealth studies, which have reported compliance rates of 80% to 95% [25,28-31]. Although 1 study reported using automated phone calls to improve compliance [31], others did not report on the level of encouragement participants were given to transmit data daily. Our study used a *three strikes* policy and, beyond that, noncompliant patients were not reminded to perform measurements. An automated system may have improved compliance and, thus, the overall results.

Patients with ACS in the intervention arm were more likely to complete CR, which is consistent with previous studies [32]. Although the intervention did not directly encourage patients to attend, it is hypothesized that the daily routine of taking measurements and the educational notifications of TCC helped engage patients and promote self-care. Thus, the benefits of TCC may have been amplified by the benefits of attending a full course of CR.

A similar principle may explain the improvement in medication adherence observed in TCC; that is, that patient education and increased engagement reinforced the importance of medication adherence in the management of their cardiac condition. It is also hypothesized that the daily requirement to measure BP was a memory trigger for taking medications.

Thus, the reduction in cardiac readmissions observed at 6 months is likely a consequence of a multifactorial mechanism. Deteriorations in the patients’ physical condition were identified and managed in the outpatient sector, and improved self-care leading to higher engagement with CR and medication adherence is likely to have made such deteriorations less likely to occur in the first place. There was no significant reduction in the incidence of 30-day readmissions, suggestive of a medium- to long-term benefit of the intervention rather than an immediate one.

The participants generally found TCC easy to use, which likely improved app compliance. Although the app did not function for 2 participants (2/164, 1.2%), all other technical issues were remedied during the trial, and no discontinuations because of technical issues occurred. The app design was optimized for older adult patients with features such as large buttons and graphical displays. The use of Bluetooth synchronization eliminated the need for manual data entry, thereby easing the work burden on the patients. In the cardiovascular mHealth space, usability data have generally been underreported; thus, there is minimal scope for comparison with other apps.

During the trial itself, the costs incurred outweighed the costs of cardiac readmissions saved. The primary contributor to this were the constant staffing costs incurred regardless of patient load. For example, at the commencement of the trial, when a small number of enrollments had occurred, and at the end of the trial, after recruitment had ceased because of COVID-19, staffing costs were incurred at the same rate as during the peak of the trial.

The 12-month cost-effectiveness model demonstrated that costs saved will exceed costs incurred when >237 patients are recruited. There are several assumptions in this model; however, they generally underestimate the cost-effectiveness of TCC. For example, it was assumed that the rate of alerts, medical consultations, and readmissions would continue unchanged from months 6-12 of a participant’s enrollment. This might not be the case if the participant’s condition stabilized, and the readmission rates compared with the control group could potentially fall further. It was also assumed that the time taken to perform certain duties by staff would remain constant when, realistically, this should reduce as the staff members become more experienced. The cost-effectiveness models also did not consider the impact of noncardiac readmissions, which were not shown to be significantly different between the intervention and control arms in this study. Ultimately, the results of a larger RCT of this program will further inform cost-effectiveness models. For cardiovascular mHealth models, only 1 cost-effectiveness analysis has been published; however, it was in the context of an SMS text messaging intervention [33] rather than a telemonitoring system.

This study is limited by its relatively small sample size and the loss of data for several physical parameters, both of which were influenced by the COVID-19 pandemic. Thus, few conclusions can be drawn on several end points, including anthropometric measurements, 6-minute walk distance, and low-density lipoprotein cholesterol. However, because of the randomized nature of the trial, participants in both arms were equally affected by the pandemic, and the end points of 30-day and 6-month readmissions, as well as medication adherence, were not affected disproportionately in either arm. The generalizability of the results should be considered with caution. The intervention was only offered to participants who were smartphone owners. If a family member offered to share the use of their own phone for the study, this was not permitted. In the HF cohort, where the mean age was 79 years, smartphone ownership rates were low (41/224, 18.3%), thus limiting enrollment. It is not known whether similar results would have been achieved if patients lacking smartphones were provided with them. However, it is anticipated that smartphone ownership rates will continue to increase in all age groups; thus, future studies may enroll a higher proportion of older adult patients.

Whether the positive outcomes identified in the TCC study have continued ongoing long-term benefits after the completion of the trial remains unknown. There are limited data regarding residual benefits of mHealth interventions, although 1 study has reported improved BMI in patients 4 years after concluding the intervention, suggesting that learned behaviors may continue in the long term [34]. As most participants in this study consented to long-term data linkage analysis, it is possible that this question can be addressed in the future. Furthermore, a large, multicenter RCT of a modified TCC program powered for clinical end points is scheduled to commence in 2021. The primary end point will be unplanned hospital readmissions at 6 months, the participants will be followed for 12 months, and an additional SMS text messaging arm will be used for patients who own a mobile phone that is not capable of operating the new app.

### Conclusions

The TCC program is a novel and innovative model of care based on a smartphone app that facilitates telemonitoring and patient education. The system was demonstrated to be safe, feasible, patient-friendly, and cost-effective when applied to patients with ACS and HF at the time of discharge. Clinical benefits were observed regarding the rate of cardiac and all-cause readmissions, medication compliance, and CR completion. These results are promising, but confirmation with a larger trial is necessary before implementing widespread adoption of the model.

## Appendix

Multimedia Appendix 1 App user evaluation questionnaire.

Multimedia Appendix 2 Methods for the 12-month cost-effectiveness model.

Multimedia Appendix 3 Medications taken at baseline for patients in the intervention and control arms.

Multimedia Appendix 4 Summary of alerts generated and their designation after investigation by the monitoring team, alert classification including examples, and summary of the causes of total and clinically significant alerts received by patients with acute coronary syndrome or heart failure.

Multimedia Appendix 5 Physical parameters at follow-up.

Multimedia Appendix 6 Quality of life and Patient Activation Measure results.

Multimedia Appendix 7 Monitoring duties.

Multimedia Appendix 8 CONSORT-eHEALTH checklist (V 1.6.1).

## Abbreviations

ACS: acute coronary syndrome
BP: blood pressure
CR: cardiac rehabilitation
ED: emergency department
GP: general practitioner
HF: heart failure
HR: hazard ratio
MGL: Morisky–Green–Levine 4-item medication compliance
mHealth: mobile health
RCT: randomized controlled trial
TCC: TeleClinical Care
TIM-HF: Telemedical Interventional Monitoring in Heart Failure
