# Supplementary material for: A Smartphone-Based Model of Care to Support Patients With Cardiac Disease Transitioning From Hospital to the Community (TeleClinical Care): Pilot Randomized Controlled Trial
Source: JMIR Mhealth Uhealth. 2022 Feb 28;10(2):e32554. doi: 10.2196/32554 (PMC8922139; doi:10.2196/32554)
Supplement: Multimedia Appendix 1 [file mhealth_v10i2e32554_app1.docx]

*THE UNIVERSITY OF NEW SOUTH WALES and PRINCE OF WALES HOSPITAL*

### *TOTAL CARDIAC CARE* USER EXPERIENCE QUESTIONNAIRE

Thank you for taking part in our study, we hope that you enjoyed participating in the *Total Cardiac Care* program!

This questionnaire helps us to evaluate your experience of the *Total Cardiac Care (TCC)* app. It also gives you a chance to provide us with valuable feedback about your experience so far.

Please take your time to carefully read and answer each question. Remember there are no right or wrong answers and your responses to this questionnaire will not affect your participation in the study in any way.

It will take approximately 25 minutes to complete. We thank you for your time.

**Please enter your name**

**__________**

**YOUR EXPERIENCE**

This section relates to your experience while using the *TCC app*.

1. **What did you like the most about the *TCC* app, and why?**

______________________________________________________________________________________________________________________________________________________________________________________________________________________________________

1. **What did you like least about the *TCC* app, and why?**

_______________________________________________________________________________________________________________________________________________________________________________________________________________________________________

1. **Do you feel like the *TCC* app benefited you in any way?**

Yes [ ]

No [ ] *If No, skip to question 4*

**3.1 If Yes: In what ways do you feel that the *TCC* app has benefited you?**

_______________________________________________________________________________________________________________________________________________________________________________________________________________________________________

____________________________________________________________________________

**YOUR OVERALL EXPERIENCE**

1. **If you had to describe your experience of using the *TCC* app in a few words, what would you say?**

__________________________________________________________________________________________________________________________________________________________

_____________________________________________________________________________

_____________________________________________________________________________

1. **Do you have any other comments or feedback?**

_________________________________________________________________________________________________________________________________________________________

_____________________________________________________________________________

____________________________________________________________________________

| **The section below is about your overall impression of the *TCC* app** | | | | | | |
| --- | --- | --- | --- | --- | --- | --- |
| **Please indicate the extent to which you agree with the following statements.**  **Circle the box that best represents your opinion.** Remember there are no right or wrong answers. | | | | | | |
|  |  | Disagree | Slightly disagree | Neither agree nor disagree | Slightly agree | Agree |
| **7.1** | I found the *TCC* app user-friendly | 1 | 2 | 3 | 4 | 5 |
| **7.2** | I enjoyed using the *TCC* app to manage my cardiac condition. | 1 | 2 | 3 | 4 | 5 |
| **7.3** | I found it re-assuring to know that my measurements on the *TCC* app are reviewed by a clinician/health expert. | 1 | 2 | 3 | 4 | 5 |
| **7.4** | I think using the *TCC* app has helped improve my cardiac condition. | 1 | 2 | 3 | 4 | 5 |
| **Circle the box that best represents your opinion on the following statement.** Remember there is no right or wrong answer. | | | | | | |
|  |  | Very poor | Poor | Neither good nor bad | Good | Very good |
| **8.1** | Overall, I would rate the *TCC* app as: | 1 | 2 | 3 | 4 | 5 |

| 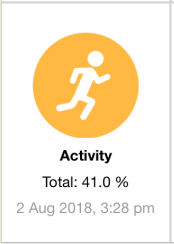  **The section below is about “physical activity” monitoring with the *TCC* app** | | | | | | | | | |  |
| --- | --- | --- | --- | --- | --- | --- | --- | --- | --- | --- |
| **For each observation, circle the number that best describes the way YOU think while monitoring the activity with the *TCC* app.** Please read the statements on each end of the scale carefully before answering. Remember there are no right or wrong answers. | | | | | | | | | | |
|  | **Statement** |  | | | | | | | **Statement** | |
| **9.1** | I found it difficult to use | 1 | 2 | 3 | 4 | 5 | 6 | 7 | I found it easy to use | |
| **9.2** | It was not useful for monitoring my physical activity | 1 | 2 | 3 | 4 | 5 | 6 | 7 | It was very useful for monitoring my physical activity | |
| **9.3** | The app did not accurately monitor my physical activity | 1 | 2 | 3 | 4 | 5 | 6 | 7 | The app accurately monitored my physical activity | |
| **9.4** | It did not influence my levels of physical activity | 1 | 2 | 3 | 4 | 5 | 6 | 7 | It significantly influenced my levels of physical activity | |
| **9.5** | I think the “activity” monitoring feature of the *TCC* app can be improved | 1 | 2 | 3 | 4 | 5 | 6 | 7 | I do not think the “activity” monitoring feature of the *TCC* app needs to be improved | |

- 1. **Do you have any other comments or feedback regarding the “physical activity” monitoring feature of the *TCC* app?**

_________________________________________________________________________________________________________________________________________________________

_____________________________________________________________________________

____________________________________________________________________________


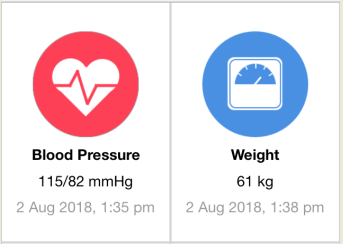


| **The section below is about the “weight” measurement with the *TCC* app** | | | | | | | | | |  |
| --- | --- | --- | --- | --- | --- | --- | --- | --- | --- | --- |
| **For each observation, circle the number that best describes the way YOU think while measuring and monitoring your weight with the *TCC* app.** Please read the statements on each end of the scale carefully before answering. Remember there are no right or wrong answers. | | | | | | | | | | |
|  | **Statement** |  | | | | | | | **Statement** | |
| **10.1** | I found it difficult to use | 1 | 2 | 3 | 4 | 5 | 6 | 7 | I found it easy to use | |
| **10.2** | It was not useful for monitoring my weight | 1 | 2 | 3 | 4 | 5 | 6 | 7 | It was very useful for monitoring my weight | |
| **10.3** | It did not have any influence on my diet | 1 | 2 | 3 | 4 | 5 | 6 | 7 | It had a significant influence on my diet | |
| **10.4** | It did not have any influence on my fluid intake | 1 | 2 | 3 | 4 | 5 | 6 | 7 | It significantly influenced my fluid intake | |
| **10.5** | It did not influence my activity levels | 1 | 2 | 3 | 4 | 5 | 6 | 7 | It significantly influenced my activity levels | |
| **10.6** | It did not influence the medications I took | 1 | 2 | 3 | 4 | 5 | 6 | 7 | It significantly influenced the medications I took | |
| **10.7** | I think the “weight” measurement feature of the *TCC* app can be improved | 1 | 2 | 3 | 4 | 5 | 6 | 7 | I do not think the “weight” measurement feature of the *TCC* app needs to be improved | |

- 1. **Do you have any other comments or feedback regarding the “weight” measurement feature of the *TCC* app?**

_________________________________________________________________________________________________________________________________________________________

_____________________________________________________________________________

____________________________________________________________________________


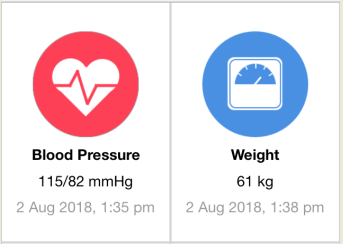


| **The section below is about the “blood pressure (BP)” measurement with the *TCC* app** | | | | | | | | | |  |
| --- | --- | --- | --- | --- | --- | --- | --- | --- | --- | --- |
| **For each observation, circle the number that best describes the way YOU think while measuring and monitoring your BP with the *TCC* app.** Please read the statements on each end of the scale carefully before answering. Remember there are no right or wrong answers. | | | | | | | | | | |
|  | **Statement** |  | | | | | | | **Statement** | |
| **11.1** | I found it difficult to use | 1 | 2 | 3 | 4 | 5 | 6 | 7 | I found it easy to use | |
| **11.2** | It was not useful for monitoring my BP | 1 | 2 | 3 | 4 | 5 | 6 | 7 | It was very useful for monitoring my BP | |
| **11.3** | It did not have any influence on my diet | 1 | 2 | 3 | 4 | 5 | 6 | 7 | It had a significant influence on my diet | |
| **11.4** | It did not have any influence on my fluid intake | 1 | 2 | 3 | 4 | 5 | 6 | 7 | It significantly influenced my fluid intake | |
| **11.5** | It did not influence my activity levels | 1 | 2 | 3 | 4 | 5 | 6 | 7 | It significantly influenced my activity levels | |
| **11.6** | It did not influence the medications I took | 1 | 2 | 3 | 4 | 5 | 6 | 7 | It significantly influenced the medications I took | |
| **11.7** | I think the “BP” measurement feature of the *TCC* app can be improved | 1 | 2 | 3 | 4 | 5 | 6 | 7 | I do not think the “BP” measurement feature of the *TCC* app needs to be improved | |

- 1. **Do you have any other comments or feedback regarding the “BP” measurement feature of the *TCC* app?**

_________________________________________________________________________________________________________________________________________________________

_____________________________________________________________________________

____________________________________________________________________________

| 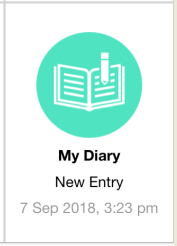  **The section below is about the “my diary, notification, and heart health tutorial” features of the *TCC* app** | | | | | | | | | | 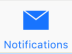  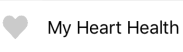 |
| --- | --- | --- | --- | --- | --- | --- | --- | --- | --- | --- |
| **For each observation, circle the number that best describes the way YOU think while using the “my diary, notification and heart health tutorial” features of the *TCC* app.** Please read the statements on each end of the scale carefully before answering. Remember there are no right or wrong answers. | | | | | | | | | | |
|  | **Statement** |  | | | | | | | **Statement** | |
| **12.1** | The “my diary” feature of the *TCC* app was not at all useful for expressing my thinking/observations about my health condition(s) | 1 | 2 | 3 | 4 | 5 | 6 | 7 | The “my diary” feature of the *TCC* app was very useful for expressing my thinking/observations about my health condition(s) | |
| **12.2** | The “notification” feature of the *TCC* app was not at all useful for regularly monitoring my blood pressure, weight and activity levels | 1 | 2 | 3 | 4 | 5 | 6 | 7 | The “notification” feature of the *TCC* app was very useful for regularly monitoring my blood pressure, weight and activity levels | |
| **12.3** | The “notification” feature did not help me to take my medications regularly | 1 | 2 | 3 | 4 | 5 | 6 | 7 | The “notification” feature significantly helped me to take my medications regularly | |
| **12.4** | The “heart health tutorial” feature of the *TCC* app was not at all useful for maintaining a healthy lifestyle | 1 | 2 | 3 | 4 | 5 | 6 | 7 | The “heart health tutorial” feature of the *TCC* app was very useful for maintaining a healthy lifestyle | |

- 1. **Do you have any other comments or feedback regarding the “my diary, notification, and heart health tutorial” features of the *TCC* app?**

_________________________________________________________________________________________________________________________________________________________

_____________________________________________________________________________

____________________________________________________________________________

| **The section below is about your opinion regarding how easy the *TCC* app was to use** | | | | | | |
| --- | --- | --- | --- | --- | --- | --- |
| **For each statement please circle the number from 1-5 that best matches whether you disagree or agree with the statement.** Please read the statements of the scale carefully before answering. Remember there are no right or wrong answers. | | | | | | |
|  | **Statement** | **Strongly**  **Disagree** | |  | **Strongly**  **Agree** | |
| **13.1** | I would like to use the *TCC* app regularly | 1 | 2 | 3 | 4 | 5 |
| **13.2** | I found the *TCC* app unnecessarily complicated | 1 | 2 | 3 | 4 | 5 |
| **13.3** | I needed technical support from another person to use *TCC* app | 1 | 2 | 3 | 4 | 5 |
| **13.4** | The features in the *TCC* app were well designed | 1 | 2 | 3 | 4 | 5 |
| **13.5** | I think most people would learn to use the *TCC* app very quickly | 1 | 2 | 3 | 4 | 5 |

| **The section below is about your attitudes to monitor your cardiac condition using *TCC* app** | | | | | | | | |
| --- | --- | --- | --- | --- | --- | --- | --- | --- |
| We would like to know whether you think continuing to use the *TCC* app to measure your BP, weight and physical activity would be good for you or not, and the reasons why. For each statement, please circle the box that is closest to your opinion. | | | | | | | | |
|  |  | Disagree strongly | Disagree | Disagree slightly | Neither agree nor disagree | Agree slightly | Agree | Agree strongly |
| **14.1** | Continuing to use the *TCC* app will improve my overall health and wellbeing | 1 | 2 | 3 | 4 | 5 | 6 | 7 |
| **14.2** | Continuing to use the *TCC* app will make me feel confident about my cardiac condition | 1 | 2 | 3 | 4 | 5 | 6 | 7 |
| **14.3** | Other people whose opinions matter to me (e.g. family, friends, doctor) think it is a good idea for me to continue to use the *TCC* app | 1 | 2 | 3 | 4 | 5 | 6 | 7 |
| **14.4** | It will not be difficult for me to continue to use the *TCC* app with other regular tasks | 1 | 2 | 3 | 4 | 5 | 6 | 7 |

| **The section below is for smokers who used the *TCC* app** | | | | | | | | |
| --- | --- | --- | --- | --- | --- | --- | --- | --- |
| **15.1** | How often did you smoke before the study? | | | | | | | If ‘once a month or week’, go to Q15.3 |
|  | ☐  Once a month | | ☐  Once a week | | | ☐  Daily | |  |
| **15.2** | How many cigarettes did you smoke, on average, per day before the study? | | | | | | |  |
|  | ☐  <1 | ☐  1-5 | | ☐  6-10 | ☐  11-20 | | ☐  >20 |  |
| **15.3** | How often do you smoke now? | | | | | | |  |
|  | ☐  Never | | ☐ ☐  Once a week Once a month | | | ☐  Daily | |  |
| **15.4** | How many cigarettes do you smoke now, on average, each day? | | | | | | |  |
|  | ☐  <1 | ☐  1-5 | | ☐  6-10 | ☐  11-20 | | ☐  >20 |  |

***Thank you for your time!***
