# Supplementary material for: A Smartphone-Based Model of Care to Support Patients With Cardiac Disease Transitioning From Hospital to the Community (TeleClinical Care): Pilot Randomized Controlled Trial
Source: JMIR Mhealth Uhealth. 2022 Feb 28;10(2):e32554. doi: 10.2196/32554 (PMC8922139; doi:10.2196/32554)
Supplement: Multimedia Appendix 2 [file mhealth_v10i2e32554_app2.docx]

**Multimedia Appendix 2: Methods for the 12-month cost effectiveness model**

Some modifications would be required to model a 12-month program, compared to the trial period. Firstly, the follow-up of the original pilot study was six months, so all healthcare costs were doubled to reflect a 12-month program. Healthcare costs included GP visits ($38.75 as per item number 23 of the Medicare Benefits Schedule) and cardiologist visits ($79.05 as per item number 116 of the Medicare Benefits Schedule). Additionally, the model required an analysis of costs saved by the reduction in cardiac readmissions. Hospitalisation costs for cardiac readmissions were provided from the New South Wales Health Activity Based Management portal. Heart failure readmissions were accounted for by the average cost of a heart failure hospitalisation ($4,450), and all other cardiac readmissions were accounted for by the average cost of an unstable angina admission ($2,083) or a myocardial infarction admission ($13,074). It was projected that a 12-month program would have double the total number of cardiac readmissions in each arm compared to the 6-month pilot study. The Mi Band would not be used as part of future TCC implementation. Since readings from the Mi Band were not monitored or acted upon, it was presumed that removing it from the health economic analysis would have no impact upon readmission rates. Licensing costs to use the web-based back-end platform (KIOLA) would be required in a real-world model, unlike in a research setting. Finally, in the 12-month model, a cardiac nurse would be remunerated based on hours worked, rather than at a fixed rate for monitoring patients and responding to alerts. The hourly wage would be provided by hospital administration. The number of hours worked would be contingent upon the number of patients being monitored. To quantify the number of hours worked, the time taken to routinely complete tasks performed by the monitoring staff was estimated by staff familiar with the TCC program and summed to determine a ‘nursing time’ per patient. This was multiplied by the hourly wage to determine the ‘nursing cost’ per patient.
