# Supplementary material for: A Smartphone-Based Model of Care to Support Patients With Cardiac Disease Transitioning From Hospital to the Community (TeleClinical Care): Pilot Randomized Controlled Trial
Source: JMIR Mhealth Uhealth. 2022 Feb 28;10(2):e32554. doi: 10.2196/32554 (PMC8922139; doi:10.2196/32554)
Supplement: Multimedia Appendix 3 [file mhealth_v10i2e32554_app3.docx]

**Multimedia Appendix 3 – Medications taken at baseline for patients in the intervention and control arms.**

| **ACS Patients** | **TCC (n=63)** | **Control (n=65)** |
| --- | --- | --- |
| **Aspirin** | 60 (95%) | 61 (94%) |
| **P2Y12** | 56 (89%) | 55 (85%) |
| **Statin** | 59 (94%) | 53 (82%) |
| **ACEI/ARB** | 42 (67%) | 39 (60%) |
| **Beta blocker** | 46 (73%) | 46 (71%) |
| **MRA** | 6 (10%) | 7 (11%) |
|  |  |  |
| **HF Patients** | **TCC  (n=18, HFPEF = 4)** | **Control  (n=18, HFPEF = 6)** |
| **Beta blocker** | 16 (89%) | 18 (100%) |
| **ACE/ARB** | 13 (72%) | 10 (56%) |
| **MRA** | 11 (61%) | 10 (56%) |
| **ARNI** | 4 (22%) | 1 (6%) |
| **Frusemide** | 10 (56%) | 12 (67%) |
| **Other diuretic** | 0 | 3 (17%) |

ACS, acute coronary syndrome; ACEI, angiotensin converting enzyme inhibitor; ARB, angiotensin receptor blocker; ARNI, angiotensin receptor neprilysin inhibitor; HF, heart failure; MRA, mineralocorticoid receptor antagonist; TCC, TeleClinical Care
