# Supplementary material for: A Smartphone-Based Model of Care to Support Patients With Cardiac Disease Transitioning From Hospital to the Community (TeleClinical Care): Pilot Randomized Controlled Trial
Source: JMIR Mhealth Uhealth. 2022 Feb 28;10(2):e32554. doi: 10.2196/32554 (PMC8922139; doi:10.2196/32554)
Supplement: Multimedia Appendix 4 [file mhealth_v10i2e32554_app4.docx]

**Multimedia Appendix 4a –** Summary of alerts generated and their designation after investigation by the monitoring team.

|  | **ACS (n=419 alerts)** | **HF (N=166 alerts)** | **Total (N=585 alerts)** |
| --- | --- | --- | --- |
| **Measurement error** | 46 (11.0%) | 14 (8.5%) | 60 (10.3%) |
| **Not of clinical significance** | 172 (41.0%) | 69 (41.5%) | 241 (41.2%) |
| **Rapid normalisation** | 144 (34.4%) | 46 (27.7%) | 190 (32.5%) |
| **Clinically significant alert** | 57 (13.6%) | 37 (22.3%) | 94 (16.0%) |

ACS, acute coronary syndrome; HF, heart failure.

**Multimedia Appendix 4b: Alert Classification including examples.**

| **Type of Alert** | **Definition** | **Example** |
| --- | --- | --- |
| Measurement error | A clearly abnormal reading that was not likely to be physiologically possible. | When the heart rate was recorded as zero, or a 75 kg patient’s weight was recorded as 150 kg. |
| Rapid normalisation | Where a measurement was repeated within 5 minutes and had normalised. | A BP reading of 170/85 mmHg (above the systolic threshold of 160 mmHg), which was 140/80 mmHg three minutes later. |
| Clinically significant alert | An alert that resulted in an additional investigation, a healthcare consultation or a change in management. | An alert for BP 180/100 mmHg which resulted in a visit to the general practitioner. |
| Not clinically significant alert | An alert that did not meet any of the above criteria. | A BP reading of 164/90 mmHg (above the threshold of 160 mmHg) but did not require any intervention. |

BP, blood pressure

**Multimedia Appendix 4c– Summary of the causes of total and clinically significant alerts received by patients with acute coronary syndrome or heart failure.**

|  | **ACS Group (all alerts)** | **ACS (CSAs only)** | **HF Group** | **HF (CSAs only)** |
| --- | --- | --- | --- | --- |
| **Tachycardia** | 101 (24%) | 18 (31%) | 52 (31%) | 11 (30%) |
| **Bradycardia** | 3 (1%) | 1 (2%) | 6 (4%) | 0 |
| **Hypertension** | 143 (34%) | 21 (37%) | 27 (16%) | 2 (5%) |
| **Hypotension** | 109 (26%) | 13 (23%) | 63 (38%) | 13 (35%) |
| **Weight Gain** | 74 (18%) | 8 (14%) | 28 (17%) | 13 (35%) |

ACS, acute coronary syndrome; CSA clinically significant alerts
