# Supplementary material for: A Smartphone-Based Model of Care to Support Patients With Cardiac Disease Transitioning From Hospital to the Community (TeleClinical Care): Pilot Randomized Controlled Trial
Source: JMIR Mhealth Uhealth. 2022 Feb 28;10(2):e32554. doi: 10.2196/32554 (PMC8922139; doi:10.2196/32554)
Supplement: Multimedia Appendix 5 [file mhealth_v10i2e32554_app5.docx]

**Multimedia Appendix 5 – Physical Parameters at follow-up.**

| **Parameter** | **Timepoint** | **TCC** | **Control** | **Statistical Analysis** |
| --- | --- | --- | --- | --- |
| **Systolic BP (mmHg)** | Baseline | 119 ± 18 (n=81) | 121 ± 18 (n=83) | Significant increase in BP at follow-up in both groups compared to baseline (*P* = .01), but no difference between groups. |
|  | Follow Up | 126 ± 18 (n=65) | 125 ± 20 (n=28) |  |
| **Weight (kg)** | Baseline | 85.0 ± 16.8 (n=81) | 87.8 ± 22.3 (n=83) | No time or group-by-time interaction. |
|  | Follow Up | 84.1 ± 16.9 (n=65) | 88.5 ± 17.2 (n=29) |  |
| **Waist circumference (cm)** | Baseline | 100 ± 13 (n=79) | 104 ± 16 (n=82) | No time or group-by-time interaction. |
|  | Follow Up | 101 ± 12 (n=27) | 107 ± 13 (n=24) |  |
| **6-minute walk distance (m)** | Baseline | 385 ± 119 (n=65) | 353 ± 124 (n=64) | 6MWD increased significantly in both groups (*P* = .004). There was no difference between the two groups. |
|  | Follow Up | 469 ± 114 (n=25) | 463 ± 125 (n=21) |  |
| **Low-density lipoprotein cholesterol (LDL-C, mmol/L)** | Baseline | 2.33 ± 0.94 (n=74) | 2.26 ± 1.05 (n=79) | LDL-C was significantly reduced in both groups at follow-up (*P* = .002), but no difference between the two groups was observed. |
|  | Follow Up | 1.81 ± 0.64 (n=39) | 1.76 ± 0.75 (n=41) |  |
