# Supplementary material for: A Smartphone-Based Model of Care to Support Patients With Cardiac Disease Transitioning From Hospital to the Community (TeleClinical Care): Pilot Randomized Controlled Trial
Source: JMIR Mhealth Uhealth. 2022 Feb 28;10(2):e32554. doi: 10.2196/32554 (PMC8922139; doi:10.2196/32554)
Supplement: Multimedia Appendix 6 [file mhealth_v10i2e32554_app6.docx]

**Multimedia Appendix 6 – Quality of life and Patient Activation Measure (PAM) results.**

|  | **TCC** | **Control** | Statistical Analysis |
| --- | --- | --- | --- |
| **Patient Activation Measure** | | |  |
| **Baseline** | 64.5 ± 14.7 (n=78) | 63.0 ± 15.9 (n=81) | PAM increased over time in both groups (*P* = .02), but there was no group-by-time interaction (*P* = .40). |
| **Follow Up** | 70.2 ± 14.7 (n=73) | 65.7 ± 15.1 (n=70) |  |
| **EQ-5D Quality of Life (self-reported score out of 100)** | | | |
| **Baseline** | 66.7 ± 18.9 (n=80) | 63.1 ± 21.5 (n=83) | The QOL score increased in both groups over time (*P*< .001) but there was no group-by-time interaction (*P* = .40). |
| **Follow Up** | 78.2 ± 15.5 (n=76) | 75.7 ± 14.5 (n=74) |  |
| **EQ-5D index score** | | | |
| **Baseline** | 0.84 ± 0.17 (n=80) | 0.80 ± 0.17 (n=83) | No time or group-by-time interaction. |
| **Follow Up** | 0.88 ± 0.12 (n=76) | 0.85 ± 0.12 (n=74) |  |
