# Supplementary material for: A Smartphone-Based Model of Care to Support Patients With Cardiac Disease Transitioning From Hospital to the Community (TeleClinical Care): Pilot Randomized Controlled Trial
Source: JMIR Mhealth Uhealth. 2022 Feb 28;10(2):e32554. doi: 10.2196/32554 (PMC8922139; doi:10.2196/32554)
Supplement: Multimedia Appendix 7 [file mhealth_v10i2e32554_app7.docx]

**Multimedia Appendix 7 – Monitoring Duties**

| **Duty** | **Total Number in the Pilot Trial** | **Number per patient (6 months)** | **Number per patient (projected 12 months)** | **Time per event (minutes)** | **Total time required per patient (projected 12 months)** |
| --- | --- | --- | --- | --- | --- |
| **Enrolment** | 81 | 1 | 1 | 60 | 60 |
| **Alert review** | 565 | 7.2 | 14.4 | 5 | 72 |
| **Phone calls to patients** | 179 | 2.2 | 4.4 | 5 | 22 |
| **Calls to healthcare professionals** | 241 | 3.0 | 6.0 | 20 | 120 |
| **Technical support** | 118 | 1.5 | 2.9 | 30 | 87 |
| **Other phone calls (e.g. compliance checks)** | 105 | 1.3 | 2.6 | 10 | 26 |
| **Total** |  |  |  |  | **387** |
